# Soluble Human Leukocyte Antigen-G5 Activates Extracellular Signal-Regulated Protein Kinase Signaling and Stimulates Trophoblast Invasion

**DOI:** 10.1371/journal.pone.0076023

**Published:** 2013-10-01

**Authors:** YiFan Guo, Cheuk-Lun Lee, Kam-Hei So, Jing Gao, William S. B. Yeung, YuanQing Yao, Kai-Fai Lee

**Affiliations:** 1 Department of Obstetrics and Gynaecology, Chinese PLA General Hospital, Beijing, China; 2 Department of Obstetrics and Gynaecology, The University of Hong Kong, Hong Kong SAR, China; 3 Centre for Reproduction, Development and Growth, The University of Hong Kong, Hong Kong SAR, China; VU University Medical Center, The Netherlands

## Abstract

Soluble human leukocyte antigen-G (HLA-G) is a non-classical class Ib HLA molecule that is secreted from blastocysts. Soluble HLA-G modulates the immune tolerance of the mother and can be used as a prognostic factor for the clinical pregnancy rate. However, the underlying mechanism of how soluble HLA-G5 affects pregnancy remains largely unknown. We hypothesized that soluble HLA-G5 promotes successful implantation and pregnancy by modulating trophoblast invasion through receptor binding and activation of extracellular signal-regulated protein kinase (ERK) signaling pathway. Recombinant HLA-G5 protein over-expressed in *E. coli* BL21 was purified to near homogeneity. We studied the expression of HLA-G5 and its receptors, the leukocyte immunoglobulin-like receptor subfamily B1 (LILRB1) and killer cell immunoglobulin-like receptor 2DL4 (KIR2DL4), in primary trophoblasts and trophoblastic (JAr and JEG-3) cell lines by florescence-labeled HLA-G5. HLA-G5 was detected in the primary trophoblasts and JEG-3 cells. The LILRB1 and KIR2DL4 receptors were expressed in both primary trophoblasts and trophoblastic cell lines. HLA-G5 stimulated cell invasion (p<0.05) and increased urokinase (uPA) and matrix metalloproteinases (MMPs) transcripts and their activity (p<0.05) in trophoblastic cells. HLA-G5 activated the ERK signaling pathway and induced ERK1/2 phosphorylation in the trophoblastic cell lines. Addition of ERK inhibitors (U0126 and PD98059) nullified the stimulatory effect of HLA-G5 on trophoblastic cell invasion. Taken together, HLA-G5 induced trophoblast invasion by binding to KIR2DL4 and LILRB1, by increasing uPA and MMPs expressions and by activating the ERK signaling pathway.

## Introduction

Trophoblast invasion plays an important role in embryo implantation and placentation. During implantation, the invasive trophoblast interacts with maternal decidual cells enabling the formation of the spiral arteries that supply the fetus during its development [1]. Although the trophoblast is semi-allogeneic and should elicit a maternal immune response [2], it does not express the classical human leucocyte antigen (HLA) class Ia and II, but rather the non-classical HLA class Ib molecules that confers maternal immunotolerance to the cells during pregnancy [3]–[6].

Among the unique HLA class Ib members, HLA-G was the first to be isolated from human extra-villous trophoblastic cell membranes [7]. HLA-G is thought to protect the trophoblast from attack by the decidual natural killer (NK) cells, macrophages and cytotoxic T cells by binding to their receptors such as the leukocyte immunoglobulin-like receptor subfamily B1 (LILRB1) and the killer cell immunoglobulin-like receptor 2DL4 (KIR2DL4) [8]. HLA-G triggers cytokine secretion, including interleukin (IL)-10, tumor necrosis factor (TNF)-α and interferon (IFN)-γ from decidual leukocytes, which contributes to placental remodeling [9]. HLA-G also inhibits cytotoxicity leading to apoptosis of the decidual leukocytes in pregnancy complications [5], [10]–[11].

Seven HLA-G isoforms can be generated from the alternative splicing of the HLA-G mRNA [12]–[13]. Four of the isoforms are membrane-bound (HLA-G1, G2, G3 and G4) and three of them are secretory (soluble HLA-G5, G6 and G7) [14]. The full-length soluble HLA-G5 has three domains, namely α1, α2 and α3, whereas HLA-G6 lacks the α2 domain and HLA-G7 lacks α2 and α3 domains [15]. The structure of HLA-G5 is similar to the classical HLA Class I molecules and can bind to the decidual leukocytes [16]. HLA-G5 is well known for its role in immune tolerance, whether it has a direct effect on trophoblast function remains unclear.

We hypothesized that HLA-G5 regulates trophoblast invasion, which in turn modulates embryo implantation and placentation. In this study we produced and characterized the HLA-G5 recombinant protein and studied the role of the HLA-G5 protein in trophoblast invasion. We examined the receptor(s) and mechanism(s) mediating the biological effects of HLA-G5.

## Materials and Methods

### Ethics Statement

The protocol in this study was approved by The Institutional Review Board of the University of Hong Kong/Hospital Authority Hong Kong West Cluster. Written consent was obtained from women before undergoing surgical termination of pregnancy and collection of placental villi samples for research.

### Recombinant HLA-G5 expression

HLA-G5 complementary DNA was prepared by reverse transcription of total RNA of the human choriocarcinoma JEG-3 cells expressing HLA-G protein [17]. Briefly, total RNA was purified using the QuickPrep RNA extraction kit (GE Healthcare, Salt Lake City, UT, USA) and reverse transcribed with the TaqMan reverse transcription reagent kit (Applied Biosystems, Foster City, CA, USA). The cDNA was then amplified using PCR with HLA-G5 primers, G5_F: 5′-ggaattccatATGGTGGTCATGGCACCCCGAACCCTCTTC-3′ and G5_R: 5′-cgcggatccTTAAAGGTCTTCAGAGAGGCTCCTGCTTTCC-3′ (the restriction sites used for cloning are underlined). The PCR conditions were 95°C for 5 minutes, 35 cycles at 95°C for 30 seconds, 57°C for 30 seconds, 72°C for 1 minute, followed by 72°C for 7 minutes. The PCR amplicon was digested with *Bam*HI and *Nde*I and cloned into a pET-15b vector (Novagen, Darmstadt, Germany). The recombinant plasmid was transformed into *E. coli* BL21 (Novagen) for the expression studies. The *E. coli* were treated with 0.5 mM isopropyl-β-D-thiogalactoside at 30°C for 3 hours to over-express the recombinant HLA-G5 protein. The expressed proteins were sequestered in the inclusion bodies of *E. coli*, and were extracted and purified using the Protein Refolding kit (Novagen). The purified protein was dialyzed in renaturing buffer (20 mM Tris-HCl, pH 8.5, with 1 mM reduced glutathione and 0.2 mM oxidized glutathione) and insoluble proteins were removed by a 0.22 µm filter. Bacterial endotoxin, which is known to induce cytokine secretion from animal cells, was removed using the Detoxi-Gel™ Endotoxin Removing Gel (Thermo Fisher Scientific Inc., Rockford, USA). The recombinant HLA-G5 protein was confirmed by Western blotting using the 5A6G7 antibody (Abcam, Cambridge, MA, USA) and by tandem mass spectrometry (MS/MS, Proteomic Laboratory for System Biology Research, HK).

### Trophoblastic cell lines and cell culture

Human choriocarcinoma JAr and JEG-3 cell lines (ATCC, Manassas, VA, USA) were cultured in RPMI-1640 and DMEM/F12 media (Sigma), respectively, supplemented with 10% heat-inactivated fetal bovine serum, 1% glutamic acid and 1% penicillin/streptomycin. The cells were cultured at 37°C in air with 5% CO_2_.

### Isolation of human primary trophoblasts

Placental villi samples were obtained from women undergoing surgical termination of pregnancy in the first trimester. Primary human trophoblasts were isolated as previously described [18]–[19]. Briefly, the collected placental villi were minced and digested in 0.25% trypsin for 15 minutes with constant shaking. Single cells were isolated by filtration through 100 µm and 40 µm filters (BD Bioscience, Bedford, MA, USA) and centrifuged with Lymphoprep (GE Healthcare, Uppsala, Sweden) at 710 g for 20 minutes. Cells were collected and cultured on a fibronectin-coated plate overnight. The adherent cells were harvested for use in the next experiments.

### Western blotting and immunostaining

The expression of HLA-G5 and receptors in trophoblasts was analyzed by Western blotting and immunostaining. For Western blotting, Cytobuster™ protein extraction reagent (Merck, Darmstadt, Germany) was used for cell lysis. Soluble proteins were separated by 10% SDS-PAGE and transferred to a PVDF membrane. Primary antibodies against HLA-G5 (5A6G7, Abcam, MA, USA), KIR2DL4 (mAb 33, Abcam) and LILRB1 (HP-F1, Abcam) were used at a dilution of 1∶1000. The anti-β-actin antibody (1∶5000) (Sigma) was used as the loading control. Recombinant HLA-G5 protein was used as the positive control, and the nature killer cell line (NK92mi) was used as the control for the detection of KIR2DL4 and LILRB1 proteins.

For the immunostaining assay, cultured cells were fixed in 4% paraformaldehyde (Sigma) at 4°C for 15 minutes and permeabilized with 0.1% Triton X-100 (Sigma) in blocking solution (3% donkey serum in PBS) for 20 minutes. The cells were blocked with 3% donkey serum in PBS at room temperature for 1 hour and then incubated at 4°C overnight with the primary antibodies (1∶1000 in blocking solution) against HLA-G5, KIR2DL4 and LILRB1. After washing five times with PBST (0.05% Triton X-100 in PBS), the cells were incubated with Alexa Fluor 488 donkey anti-mouse IgG antibody (Invitrogen, Carlsbad, CA, USA) for 1 hour, then washed and examined under a fluorescence microscope (Nikon, Japan).

### HLA-G5 Binding assay

Flow cytometry was used to assess the binding of HLA-G5 to the cells as previously described [20]. Briefly, recombinant HLA-G5 protein was labeled using the Alexa Fluor 488 microscale protein labeling kit (Molecular Probes, Carlsbad, CA, USA). Pre-labeled HLA-G5 protein (1 µg/mL) was incubated with the JAr and JEG-3 cells (5×10^5^ each) for 1 hour. For the antibody blocking group, pre-labeled HLA-G5 protein was mixed with the 5A6G7 antibody in a molar ratio of 1:5 at room temperature for half an hour before incubation with the cells. After washing, the fluorescence signal was detected using a BD FACSCantoII flow cytometer (BD Bioscience, San Jose, CA, USA) equipped with an argon laser (488 nm wavelength with a 525 nm band pass filter). The results were analyzed using the FlowJo software (Tree Star Inc., Ashland, OR, USA).

### Proliferation assay

The proliferation of the JAr and JEG-3 cells were measured with the fluorometric CyQUANT NF Cell proliferation assay Kit (Invitrogen) in accordance with the manufacturer’s instructions. Cells at a density of 6000 cells/well were treated with different concentrations of HLA-G5 recombinant protein (0, 0.1, 1, 10 µg/mL) for 24 hours before incubating with the dye binding solution for 45 minutes. The fluorescence intensity was measured using a fluorescence microplate reader with excitation at 485 nm and emission detection at 530 nm. The results were presented as the percentage of fluorescence intensity relative to the untreated group.

### Migration assay

The CytoSelect™ 96-well Cell Migration Assay Kit (Cell Biolabs Inc., CA, USA) was used to study the trophoblast migration. Briefly, 100 µL of the cell suspension was placed in the upper chamber at a density of 5×10^5^ cells/well, and treated with HLA-G5 (0, 0.1, 1 µg/mL) for 22 hours. The migratory cells at the bottom of the polycarbonate membrane of the chamber were dissociated, lysed and then detected using CyQuant® GR Dye. The results were presented as the percentage of fluorescence intensity relative to the untreated group.

### Invasion assay

The invasiveness of trophoblasts was measured in a Matrigel pre-coated invasion chamber (BD BioCoat™ Matrigel™ Invasion Chamber, BD Bioscience) as previously described [21]. Briefly, JAr and JEG-3 cell suspensions were placed in the inserts at a density of 2×10^5^ cells/mL and treated with different concentrations of HLA-G5 recombinant protein (0, 0.1, 1 µg/mL) for 24 hours. The primary trophoblasts were seeded at a density of 5×10^6 ^cells/mL and were treated with HLA-G5 for 5 days. The invasive cells at the bottom of the chamber membrane were stained with 0.1% crystal violet (Millipore, Billerica, CA, USA) and quantified using a fluorescence microplate reader with excitation at 480 nm and emission at 520 nm. The results were presented as the percentage of fluorescence intensity relative to the untreated group.

### Expression of urokinase (uPA) and matrix metalloproteinases (MMPs)

Trophoblast cells were treated with different concentrations of the HLA-G5 recombinant protein (0, 0.1, 1 µg/mL) for 24 hours. The cells and the spent culture media were collected. Total RNA was isolated and used for real-time PCR analysis of uPA (TaqMan probe: Hs00170182_m1), MMP2 (TaqMan probe: Hs00901425_m1) and MMP9 (TaqMan probe: Hs00957555_m1) expressions using an ABI 7500 sequence detector (Applied Biosystems). The housekeeping 18S rRNA (TaqMan probe: Hs99999901_s1) was used as the endogenous control. The results were presented as the threshold cycle (CT) normalized with 18S and expressed relative to the untreated control. The activities of the secreted MMP-2 and -9 in the culture media were determined by gelatin zymography as previously described [22]. In addition, the activity of uPA was measured using the QIA 125 Urokinase Activity Assay kit (Calbiochem, La Jolla, CA, USA) in accordance with the manufacturer’s instructions. Briefly, the collected culture media were mixed with 50 µl of substrate solution in a 96-well microplate and incubated at 37°C for 45 minutes. Blank culture medium was used as the control. The fluorescence intensity was quantified using a fluorescence plate reader with excitation at 380 nm and emission at 460 nm. The results were presented as the percentage of fluorescence intensity relative to blank control.

### Analysis of signaling pathways involved in trophoblast invasion

Trophoblast cells were treated with 1 µg/mL recombinant HLA-G5 protein for 5, 15 and 30 minutes. Western blotting was used to detect the expression of mitogen-activated protein kinases (MAPK), extracellular signal-regulated protein kinases (ERK), phosphorylated ERK (BD Biosciences) and β-actin using their primary antibodies at a concentration of 1:1000. The results were presented as the percentage relative to the untreated control. The ERK inhibitors U0126 and PD98059 at concentrations of 10 µM were used to pre-treat the trophoblast for 30 minutes, and the cells were then incubated with HLA-G5 for 24 hours. The changes in trophoblast invasion were studied using the invasion chamber described above.

### Analysis of the role of LILRB1 and KIR2DL4 in mediating trophoblast function

Trophoblast cells were pre-treated with antibodies against KIR2DL4 or LILRB1 (in a molar ratio of 5∶1 to HLA-G5) for 1 hour and the binding of HLA-G5 onto cells was determined. The effect of HLA-G5 on invasion of cells pre-treated with antibodies was also examined.

### Data Analysis and Statistics

All data were presented as the mean ± standard error mean (SEM). Statistical analysis was performed using one-way ANOVA or t-test (for data normally distributed) or Kruskal-Wallis ranked one-way ANOVA (for data with skewed distribution). The *p*-values less than 0.05 were considered statistically significant.

## Results

### Synthesis of the HLA-G5 recombinant protein

**Figure 1:**
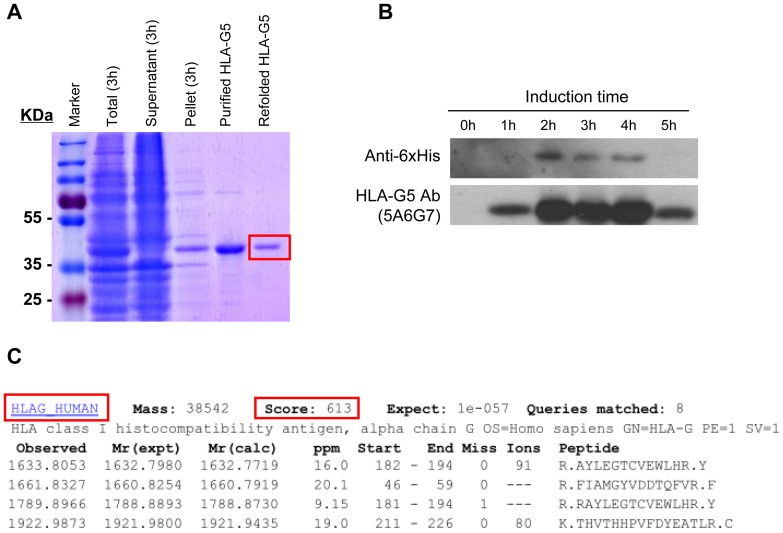
Expression and purification of HLA-G5 protein from *E. coli*. (A) Recombinant HLA-G5 protein was purified from *E. coli* BL21 cells and stained with Coomassie brilliant blue. HLA-G5 protein was expressed in total bacterial lysate after 3 hours of IPTG induction (Total, 3 hrs). The HLA-G5 protein was further purified from the inclusion bodies (pellet) of the lysate and refolded (refolded HLA-G5) in native dialysis buffer. (B) Western blot analysis of IPTG induced (0 – 5 hrs) recombinant HLA-G5 protein from the total bacterial lysate probed with His-Tag (Anti-6xHis) and HLA-G5 (5A6G7) antibodies. (C) The identity of the purified HLA-G5 protein was confirmed by mass spectrometry (MS/MS). The peptide score for the HLA-G5 protein was 613.

The *E*. coli BL21 cells treated with isopropyl-β-D-thiogalactoside over-expressed HLA-G5 proteins that were detected in the inclusion bodies (Figure 1A). The recombinant HLA-G5 proteins extracted from the inclusion bodies were purified after refolding, resulting in a single 38 kDa protein band as predicted. The protein was confirmed to be HLA-G5 by Western blotting and by mass spectrometry (peptide score was 613) (p<0.001) (Figure 1B and 1C).

### HLA-G5 bound to JAr and JEG-3 cells via specific receptors

**Figure 2:**
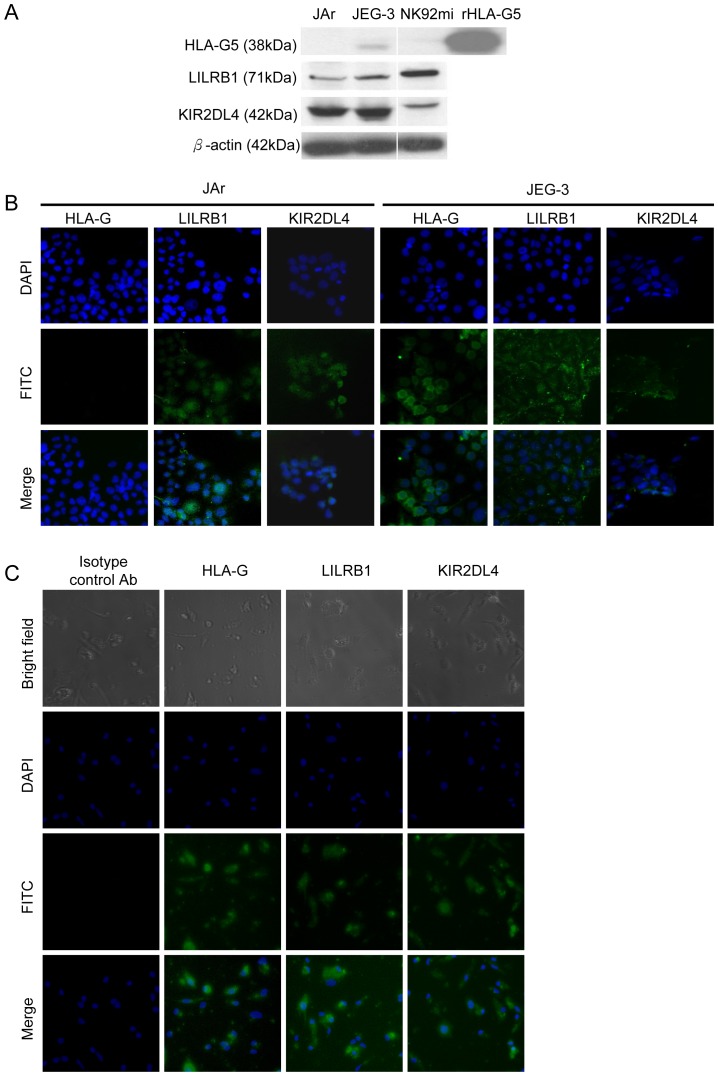
Expression of HLA-G5 and its receptors LILRB1 and KIR2DL4 in trophoblasts. ( A) Western blot analysis of the total cell lysate showed HLA-G5 was expressed in human choriocarcinoma JEG-3 cells but not in JAr or human natural killer NK92mi cells. JEG-3, JAr and NK92mi cells expressed both LILRB1 and KIR2DL4 receptors. The β-actin protein was used as loading control. (B) Immunofluorescent staining (green) showed the LILRB1 and KIR2DL4 receptors were expressed in the JAr and JEG-3 cells mainly in the cytoplasm and membrane areas. Representative images are from three sets of individual experiments. (C) Human primary trophoblastic cells stained for HLA-G5, LILRB1 and KIR2DL4 receptors. The nuclei of the cells were counterstained with DAPI. 100x Magnification.

Western blot analysis (Figure 2A) and immunostaining (Figure 2B) showed that HLA-G5 protein was expressed in JEG-3 but not in JAr or NK92mi cells. The HLA-G5 specific receptors LILRB1 and KIR2DL4 were found in all three cell lines. Similarly, HLA-G5, LILRB1 and KIR2DL4 proteins were all expressed in the human primary trophoblasts (Figure 2C).

**Figure 3:**
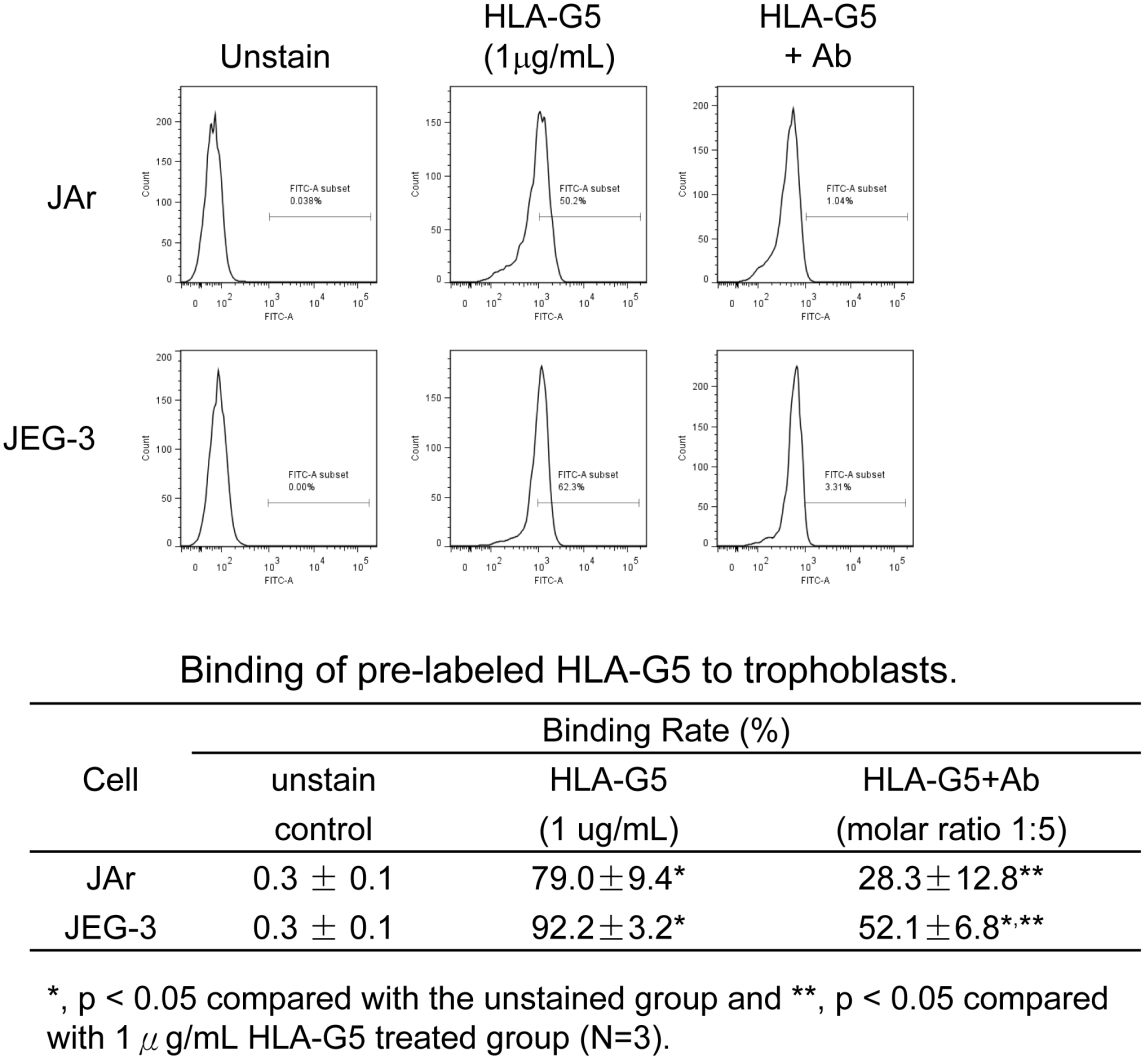
Binding of pre-labeled HLA-G5 on JAr and JEG-3 trophoblastic cells. Fluorescent-labeled HLA-G5 protein (1 µg/mL) was bound to trophoblastic cells. The percentage of the bound cells was analyzed by flow-cytometry. Treating the JAr and JEG-3 cells with 5A6G7 antibody significantly reduced the binding signal of HLA-G5 (from 79.0% to 28.3% and from 92.2% to 52.1%, respectively).

Flow cytometry analysis of labeled HLA-G5 in the trophoblastic cell lines showed that HLA-G5 (1 µg/mL) strongly bound to JAr and JEG-3 cells. HLA-G5 pre-treated with the anti-HLA-G polyclonal antibody 5A6G7 significantly reduced the binding of HLA-G5 to the JAr and JEG-3 cells (from 79.0% to 28.3% and from 92.2% to 52.1%, respectively; p<0.05) compared to the unstained HLA-G5 control group (Figure 3).

### HLA-G5 increased the invasion but not migration of trophoblasts

**Figure 4:**
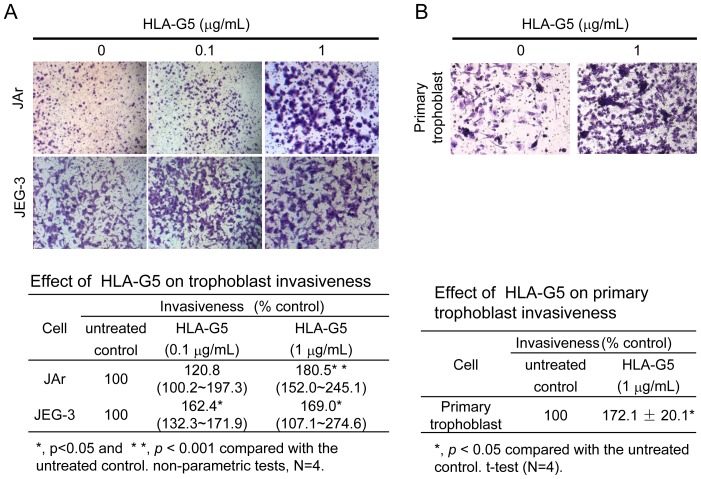
Effect of HLA-G5 on trophoblast invasiveness. (A) HLA-G5 (1 µg/mL) significantly increased the invasiveness of JAr and JEG-3 cells (100x Magnification; N = 4, p<0.05) in the trans-well invasion assay. (B) Similarly, HLA-G5 (1 µg/mL) significantly increased the invasiveness of primary human trophoblasts (100x Magnification; N = 4, p<0.05). All data are presented as the percentage of invasion relative to untreated control groups.

HLA-G5 (≤1 µg/mL) did not affect the proliferation of JAr and JEG-3 cells. However, HLA-G5 (10 µg/mL) treatment significantly reduced the proliferation of trophoblastic cells (p<0.01) compared to the untreated controls (Figure S1 in Supporting File S1). HLA-G5 (1 µg/mL) treatment increased the invasiveness of JAr cells (p<0.001) (Figure 4A) and HLA-G5 (0.1 or 1 µg/mL) increased the invasiveness JEG-3 cells (p<0.05) (Figure 4A) compared to the untreated controls. HLA-G5 treatments also increased the invasiveness of the primary trophoblasts (p<0.05) (Figure 4B). On the other hand, the migration rate of the JAr and JEG-3 cells were unchanged with HLA-G5 treatments (Figure S2 in Supporting File S1).

### HLA-G5 altered mRNA transcription and activity of uPA and MMPs

**Figure 5:**
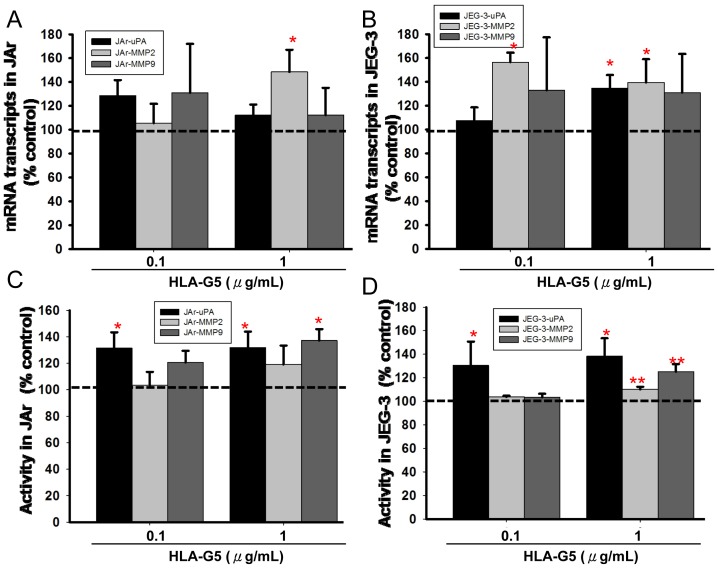
Effect of HLA-G5 on uPA/MMPs expression and activity in trophoblastic cells. Real-time PCR analysis of the uPA and MMPs (MMP2 and MMP9) transcripts in HLA-G5 treated (A) JAr and (B) JEG-3 cell lines (N = 6). In JAr cells, the expression of MMP2 transcript increased significantly after treating with HLA-G5 (*, p<0.05), whereas the uPA and MMP9 were unchanged. In JEG-3 cells, the expression of MMP2 and uPA increased significantly after treating with HLA-G5 (*, p<0.05), whereas MMP9 was unchanged. (C and D) The activity of uPA was significantly induced with HLA-G5 in both cell lines (N = 4). Similarly, the activities of MMP9 in both cell lines were significantly increased with HLA-G5 (1 µg/mL) (*, p<0.05), whereas the activities of MMP2 were significantly increased with HLA-G5 (1 µg/mL) only in the JEG-3 cells (**, p<0.01) compared to untreated controls.

HLA-G5 treatments increased the mRNA expression and activities of proteases in both the JAr and JEG-3 cells (Figure 5A & B). In the JAr cells treated with HLA-G5 (1 µg/mL), the expression of MMP2 transcript significantly increased (p<0.05), whereas uPA and MMP9 expressions were unchanged. In the JEG-3 cells, the expression of MMP2 transcript significantly increased (p<0.05) with HLA-G5 (0.1 and 1 µg/mL) treatments and the uPA transcript level also increased (p<0.05) with HLA-G5 (1 µg/mL) treatment. The expression of MMP9 transcript was not affected.

The activity of uPA was significantly induced with HLA-G5 (0.1 and 1 µg/mL) treatments in both cell lines (p<0.05) (Figure 5C). In the JAr cells, the activity of MMP9 was significantly induced with HLA-G5 (1 µg/mL) (p<0.05), whereas the MMP2 activity was unchanged (Figure S3 in Supporting File S1). In the JEG-3 cells, the MMP2 and MMP9 activities were significantly induced (p<0.01) with HLA-G5 (1 µg/mL) (Figure 5D). All the data were presented as percentages relative to the untreated control (Table S1 in Supporting File S1).

### HLA-G5 induced phosphorylation of ERKs in trophoblasts

**Figure 6:**
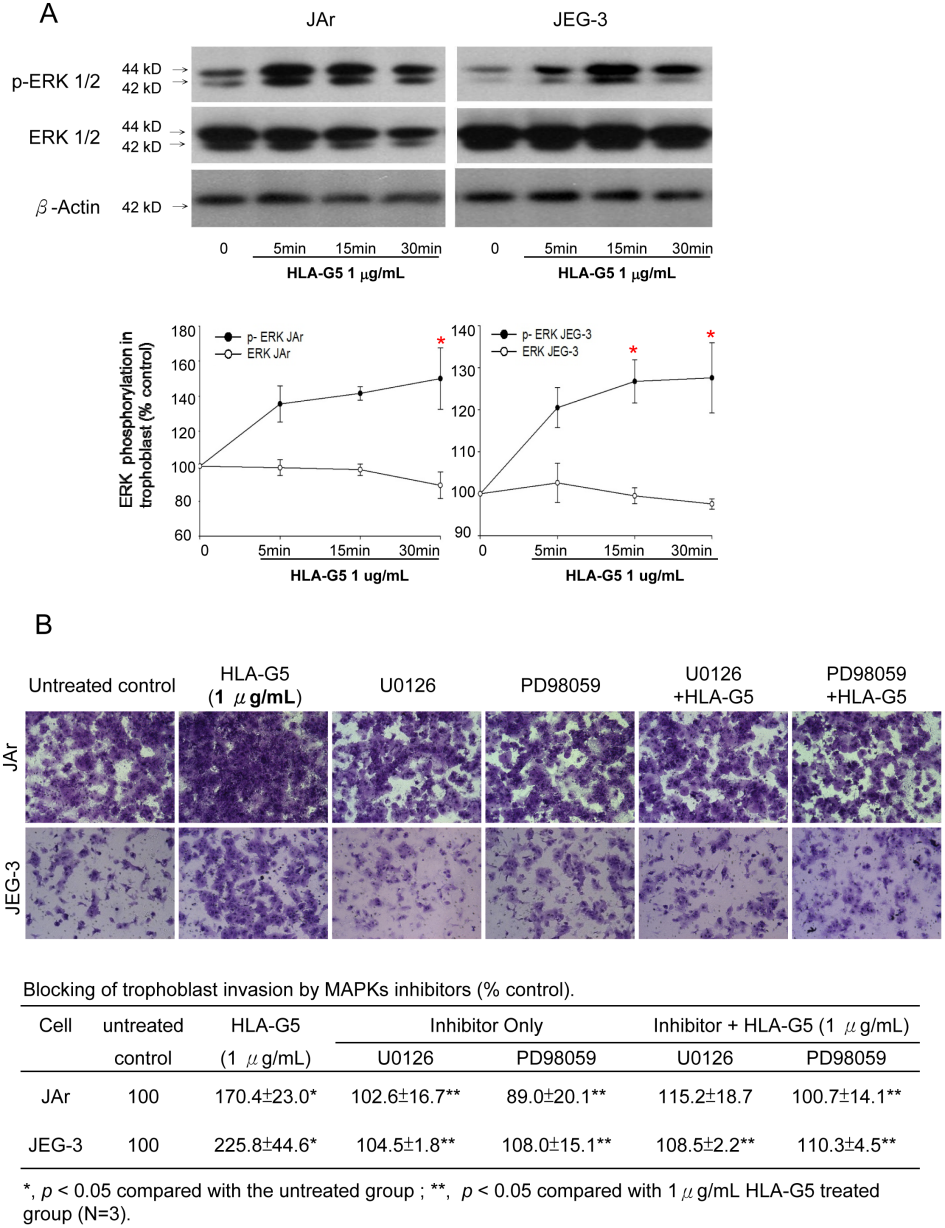
Effect of HLA-G5 on the modulation of ERK signaling pathways in trophoblast invasion. (A) HLA-G5 treatment significantly increased phosphorylated ERK expression in JAr and JEG-3 cells within 30 minutes, whereas the expression of total ERK remained mostly constant (N = 3, p<0.05) compared to untreated controls. (B) Addition of ERK inhibitors (U0126 and PD98059) reduced the stimulatory effect of HLA-G5 (1μg/mL) on the invasion of JEG-3 and JAr cells in the trans-well invasion assay compared to the control group (N = 3).

Treatment with HLA-G5 (1 µg/mL) did not affect the ERK expression. However, the phosphorylated ERK level was significantly increased in both the JAr and JEG-3 cells treated with HLA-G5 (p<0.05) compared to the untreated control (Figure 6A; Table S2 in Supporting File S1). We used the ERK inhibitors, U0126 or PD98059, to study whether HLA-G5 stimulated trophoblastic cell invasion through the ERK pathway. ERK inhibitors alone had no significant effect on cell invasion, but both inhibitors abolished the stimulatory effect of HLA-G5 (1 µg/mL) on the trophoblast invasion (p<0.05) (Figure 6B).

### KIR2DL4 and LILRB1 mediated the HLA-G5 induced trophoblast invasion

**Figure 7:**
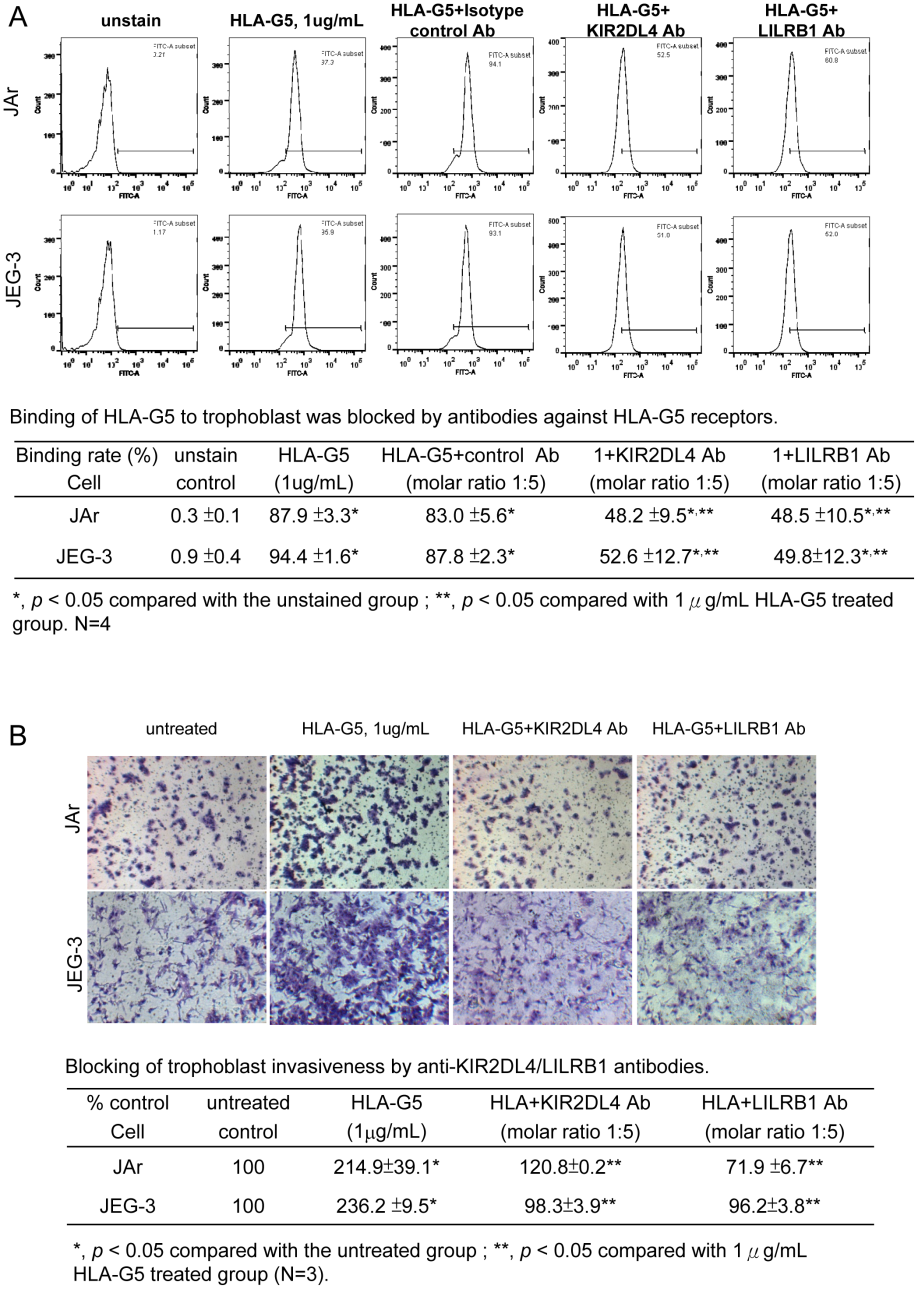
Flow cytometric analysis of the anti-KIR2DL4/LILRB1 antibody treatments on the binding of pre-labeled HLA-G5 in JAr and JEG-3 cells. (A) The binding of pre-labeled HLA-G5 in trophoblastic cells was significantly reduced after treatments with KIR2DL4 or LILRB1 antibodies (N = 4, p<0.05), whereas the isotypic antibody had no inhibitory effect on HLA-G5 binding. (B) The invasiveness of JAr and JEG-3 was significantly reduced after treatment with KIR2DL4 or LILRB1 antibodies (N = 3, p<0.05).

Treatment with anti-KIR2DL4 antibody significantly reduced the binding of HLA-G5 to the JAr and JEG-3 cells (from 87.9% to 48.2% and from 94.9% to 52.6%, respectively; p<0.05) (Figure 7A). Similarly, anti-LILRB1 antibody reduced the HLA-G5 binding to JAr and JEG-3 cells (from 87.9% to 48.5% and from 94.9% to 49.8%, respectively; P<0.05). The antibody treatments abolished the HLA-G5-induced invasiveness of JAr and JEG-3 cells. The invasiveness of JEG-3 cells was reduced from 236.2% to 98.3% by the anti-KIR2DL4 antibody and reduced to 96.2% with the anti-LILRB1 antibody. Similar results were found with JAr cells treated with these antibodies (p<0.05) (Figure 7B).

## Discussion

We demonstrated that HLA-G5 binding to KIR2DL4 and LILRB1 receptors could stimulate trophoblast invasion by increasing uPA/MMP levels. This stimulatory effect on the invasion by HLA-G5 binding to KIR2DL4 and LILRB1 was mediated via ERK activation. Trophoblast invasion is crucial to the implantation process. Insufficient invasion leads to preeclampsia, intrauterine growth restriction and spontaneous abortion, whereas excessive invasion may lead to placenta accreta [11]. HLA-G5 indirectly induces trophoblast invasion by binding to decidual leukocytes [15], [23]–[25], which produce cytokines such as IFN-γ, TNF-α, IL-1β and IL-2 that favor trophoblast invasion and placental vascularization [9], [16], [26]. In addition, HLA-G5 induces neo-expression and up-regulation of its receptors on immune cells, enhancing immune responses that favor trophoblast invasion [25]. Trophoblasts also secrete numerous cytokines including IL-1 and IL-6 that induce cell invasion [27].

In this study, HLA-G5 stimulated the invasiveness of the HLA-G positive JEG-3 cells and HLA-G negative JAr cells [17]. Because both JAr and JEG-3 cells were derived from choriocarcinoma cell lines, we also used human primary trophoblast from first trimester pregnancy. We found that HLA-G5 had a stimulatory effect on the invasion of primary trophoblasts, and a significant effect was observed after longer incubation.

A previous study reported that HLA-G5 inhibited growth factor-stimulated trophoblast (SGHPL-4 cell line) invasion, but did not affect trophoblast invasion in the absence of stimulation [28]. Our present study used a lower concentration of HLA-G, which could account for the discrepancy between the results of the reported study (100 U/mL was 100 times our concentration) and our study. The concentrations of HLA-G (0.1 – 1 µg/mL) used in our experiments were within the reported serum HLA-G levels (0.016 – 4 µg/mL) in normal pregnant women [29]. Both the JEG-3 and the SGHP-4 cell lines express similar levels of HLA-G (0.4 µg/mL) [28], [30]. High concentrations of HLA-G could inhibit trophoblast proliferation (Figure S1 in Supporting File S1) and counteract the stimulatory effect of HLA-G on cell invasion.

During the process of invasion, the trophoblast degrades and remodels the extracellular matrix of the endometrium. The trophoblast invasion during first trimester is regulated by the main proteinases, MMP2, MMP9 and uPA [31]–[32]. MMP2 plays a predominant role in the initiation of implantation. During weeks 5–9 of gestation, MMP9 production increases, which promotes trophoblast invasion [31]. uPA is involved in trophoblast invasion and also mediates spiral artery remodeling [33]. HLA-G5 enhanced the expression of these MMPs and uPA transcripts and increased their activities.

Although numerous factors are known to regulate trophoblast invasion through the activation of specific receptors, downstream signaling pathways such as the mitogen-activated protein kinase (MAPK) cascade also play an important role in trophoblast invasion [34]–[35]. The MAPKs are a large family of protein kinases (ERKs, SAPK/JNKs and p38) involved in cell cycle regulation [22], [34], [36]. Our study showed that HLA-G5 treatment activated the ERK pathway via phosphorylation of ERKs. This was further confirmed by the reversal of the HLA-G5-induced trophoblast invasion in cells pre-treated with ERK inhibitors, U0126 and PD98059. This observation is in agreement with other reports on the role of MAPK/ERK signaling pathways in trophoblast invasion [22], [37]–[38].

The HLA-G5-induced trophoblast invasion in the JAr cells, JEG-3 cells and primary trophoblast depends on the binding of HLA-G5 to the KIR2DL4 and LILRB1 receptors. Blocking these receptors with antibodies reduced the binding of HLA-G5 to the trophoblast, and abolished the stimulatory effect of HLA-G5 on trophoblast invasion. While these results confirm the involvement of the binding of HLA-G5 to KIR2DL4 and LILRB1, the existence of other HLA-G5 specific receptors on trophoblasts cannot be excluded. For example, CD160 is a HLA-G receptor found in endothelial cells [39], but it has not been reported in trophoblasts.

Some studies support the finding that decreased HLA-G production plays a role in the pathophysiology of preeclampsia by causing a decrease in maternal immune tolerance to invasive trophoblasts resulting in defective trophoblastic invasion [40]. Yet, it has been shown that first trimester serum soluble HLA-G1/G5 did not predict high-risk pregnancies in humans [41]. Therefore, a larger number of cases would be needed in order to determine whether soluble HLA-G5 could be used for prediction for these complications.

In sum, our results showed that HLA-G5 induced trophoblast invasion by increasing uPA and MMPs expression/activity, which was mediated by binding of HLA-G5 to KIR2DL4 and LILRB1 receptors and involves the ERK signaling pathway. In line with this, recent data suggested that the amount of secretory HLA-G in embryo culture medium is a sensitive and non-invasive biochemical marker for evaluating embryo quality and subsequent pregnancies [42]. Understanding the molecular mechanisms involved in HLA-G5 mediated trophoblast invasion may allow us to identify new therapeutic approaches for placental related disease including preeclampsia and placenta accreta.

## Supporting Information

Supporting File S1 **Figure S1 Effect of HLA-G5 on trophoblast proliferation.** The proliferation rates of trophoblasts were not affected by HLA-G5 (0.1 or 1 µg/mL) treatments. However, at higher a concentration of HLA-G5 (10 µg/mL), the proliferation rates of JAr and JEG-3 cells were significantly reduced compared to the untreated control (N = 8, p<0.01). The results are presented as the percentage of fluorescence intensity relative to the untreated group. **Figure S2 Effect of HLA-G5 on trophoblast migration.** The migration rates of JAr and JEG-3 trophoblasts were not affected by HLA-G5 (0.1 or 1 µg/mL) treatments (N = 8). The results are presented as the percentage of fluorescence intensity relative to the untreated group. **Figure S3 Gelatin zymography of MMP2/MMP9 activity on HLA-G5 treated trophoblastic cells.** In JAr cells, the activity of MMP9 was induced with HLA-G5 (1 µg/mL) treatment (p<0.05), whereas in the JEG-3 cells, the activities of MMP2 and MMP9 were both induced with HLA-G5 (1 µg/mL) treatment (N = 3, p<0.01). **Table S1 Changes of uPA/MMPs expression and activity in trophoblast.** In JAr cells, the expression of MMP2 increased significantly (p<0.05), whereas the uPA and MMP9 were unchanged after treatment with HLA-G5 (1 µg/mL). In JEG-3 cells, the expression of MMP2 increased significantly after HLA-G5 (0.1 and 1 µg/mL) treatments (p<0.05) but MMP9 was unchanged, and the uPA expression increased significantly with HLA-G5 (1 µg/mL) (p<0.05). The uPA activities in both cell lines were significantly induced by HLA-G5 (0.1 and 1 µg/mL) treatments (N = 4, p<0.05). Gelatin zymographic analysis (N = 3) of MMP2 and MMP9 activity in JAr cells showed the activity of MMP9 was significantly induced with HLA-G5 (1 µg/mL) (p<0.05), whereas in the JEG-3 cells, the activities of MMP2 and MMP9 were both significantly induced with HLA-G5 (1 µg/mL) (p<0.01). **Table S2 ERK phosphorylation in trophoblast.** The phosphorylated ERK was significantly induced after HLA-G5 treatment (p<0.05), whereas the expression of ERK remained mostly unchanged.
(DOC)
