# Supplementary material for: Soluble Human Leukocyte Antigen-G5 Activates Extracellular Signal-Regulated Protein Kinase Signaling and Stimulates Trophoblast Invasion
Source: PLoS One. 2013 Oct 1;8(10):e76023. doi: 10.1371/journal.pone.0076023 (PMC3787956; doi:10.1371/journal.pone.0076023)
Supplement: Supplementary file 1 [file pone.0076023.s001.doc]

Figure S1

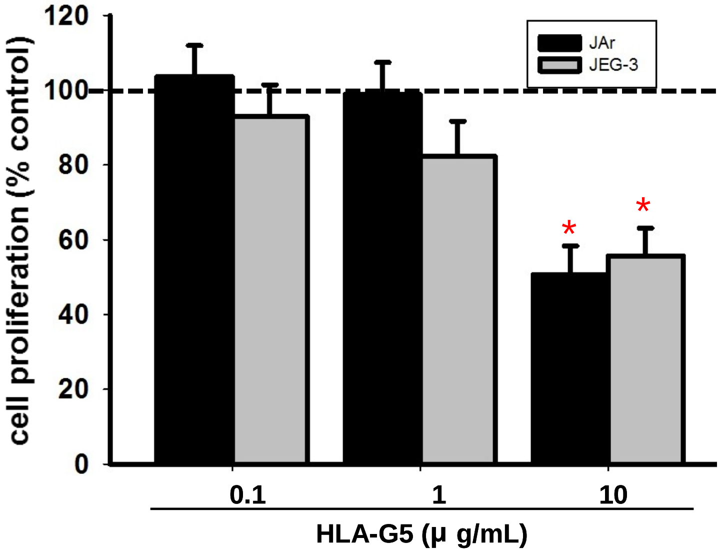

Effect of HLA-G5 on cell proliferation

Cell	Proliferation (% control)			
	ctrl	HLA-G5 (μg/mL)		
	0	0.1	1	10
JAr	100	103.6±8.3	98.8±8.5	50.7±7.6*
JEG-3	100	92.9±8.6	82.3±9.4	55.7±7.4*

*, $p < 0.05$ compared with the untreated control (N=8)

Figure S2

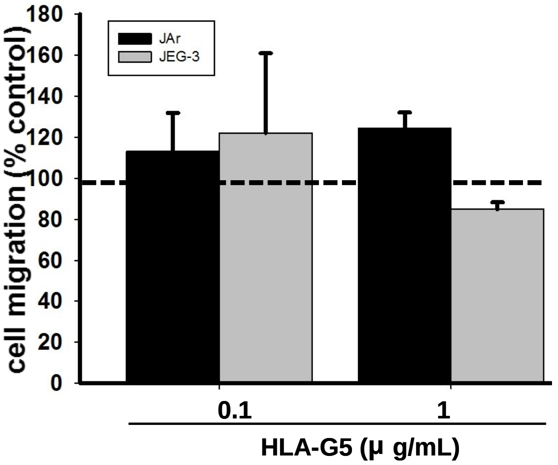

Effect of HLA-G5 on cell migration

Cell	ctrl 0	migration (% control) HLA-G5 (0.1 μg/mL)	HLA-G5 (1 μg/mL)
JAr	100	113.1±18.6	124.4±7.7
JEG-3	100	122.1±38.9	84.8±3.4

$p > 0.05$ compared with control. N=8

Figure S3

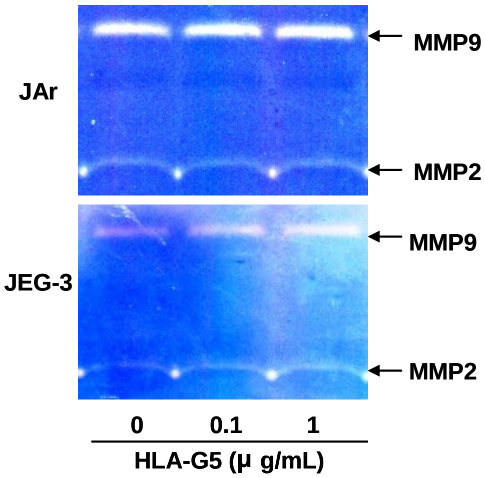

MMPs activity (% control)

Cell	MMPs activity (% control)	HLA-G5 (μ g/mL) 0	0.1	1
JAr	MMP2	100	103.4±10.1	119.1±14.3
	MMP9	100	120.6±8.8	137.1±8.6*
JEG-3	MMP2	100	103.6±1.1	110.1±2.3**
	MMP9	100	103.5±2.9	125.1±6.5**

* * , $p < 0.01$; *, $p < 0.05$ compared with the untreated control.

Table S1

uPA/MMPs mRNA transcripts (% control)

Cell	uPA/MMPs (% control)	HLA-G5 (μ g/mL) 0	0.1	1
JAr	uPA	100	128.6±13.0	112.2 ±9.0
	MMP2	100	105.6±16.2	148.5 ±18.6*
	MMP9	100	131.0±40.9	112.3±22.8
JEG-3	uPA	100	107.5±11.0	134.8 ±11.0*
	MMP2	100	156.6 ±8.0*	139.6 ±19.4*
	MMP9	100	133.0±44.4	131.0±32.4

uPA activity (% control)

uPA activity (% control)	HLA-G5 (μ g/mL) 0	0.1	1
JAr	100	131.4±11.9*	131.8±12.1*
JEG-3	100	130.4±20.3*	138.4±15.2*

*, $p < 0.05$ compared with the untreated control.

Table S2

ERK phosphorylation in trophoblast (% control)

Cell	(% control)	untreated	HLA-G5 (1 μ g/mL)		
		control	5min	15min	30min
JAr	p-ERK 1/2	100	135.6±10.4	141.6±3.9	150.0±17.6*
	ERK 1/2	100	99.1±4.5	98.0±3.3	89.0±7.6
JEG-3	p-ERK 1/2	100	120.5±4.8	126.8±5.1*	127.6±8.4*
	ERK 1/2	100	102.6±4.6	99.5±1.9	97.6±1.2

*, $p < 0.05$ compared with the untreated control. ERK 1/2 and p-ERK 1/2 were respectively normalized by β -actin (p-ERK 1/2: phosphorylated ERK 1/2). N=3
